# Constitutive and Stress-induced Expression of CCL5 Machinery in Rodent Retina

**DOI:** 10.4172/2155-9899.1000506

**Published:** 2017-05-24

**Authors:** D'Anne S. Duncan, William M. McLaughlin, Noah Vasilakes, Franklin D. Echevarria, Cathryn R. Formichella, Rebecca M. Sappington

**Affiliations:** 1 Department of Ophthalmology and Visual Sciences, Vanderbilt Eye Institute, Vanderbilt University Medical Center, USA; 2 Neuroscience Graduate Program, Vanderbilt University, USA; 3 Department of Pharmacology, Vanderbilt University School of Medicine, 11425 Medical Research Building IV, Nashville, TN 37232-0654, USA

**Keywords:** Chemokine, Glaucoma, Retina, Muller glia, Retinal ganglion cell

## Abstract

Signaling by inflammatory cytokines and chemokines is associated with neurodegeneration in disease and injury. Here we examined expression of the β-chemokine CCL5 and its receptors in the mouse retina and evaluated its relevance in glaucoma, a common optic neuropathy associated with sensitivity to intraocular pressure (IOP). Using quantitative PCR, fluorescent *in situ* hybridization, immunohistochemistry and quantitative image analysis, we found CCL5 mRNA and protein was constitutively expressed in the inner retina and synaptic layers. CCL5 appeared to associate with Müller cells and RGCs as well as synaptic connections between horizontal cells and bipolar cells in the OPL and amacrine cells, bipolar cells and RGCs in the IPL. Although all three high-affinity receptors (CCR5, CCR3, CCR1) for CCL5 were expressed constitutively, CCR5 expression was significantly higher than CCR3, which was also markedly greater than CCR1. Localization patterns for constitutive CCR5, CCR3 and CCR1 expression differed, particularly with respect to expression in inner retinal neurons. Stress-related expression of CCL5 was primarily altered in aged DBA/2 mice with elevated IOP. In contrast, changes in expression and localization of both CCR3 and CCR5 were evident not only in aged DBA/2 mice, but also in age-matched control mice and young DBA/2 mice. These groups do not exhibit elevated IOP, but possess either the aging stress (control mice) or the genetic predisposition to glaucoma (DBA/2 mice). Together, these data indicate that CCL5 and its high-affinity receptors are constitutively expressed in murine retina and differentially induced by stressors associated with glaucomatous optic neuropathy. Localization patterns further indicate that CCL5 signaling may be relevant for modulation of synapses in both health and disease, particularly in the inner plexiform layer.

## Introduction

Glaucoma is a chronic, age-related optic neuropathy, and the second leading cause of blindness worldwide [1]. Irreversible vision loss results from the degeneration of retinal ganglion cells (RGCs), whose axons form the optic nerve [2,3]. While age is the primary risk factor for glaucoma, elevated intraocular pressure (IOP) is the primary modifiable risk factor and the current target for therapy [2,3]. Although the direct cause of this RGC degeneration has yet to be determined [2-4], it shares many cellular characteristics with neurodegenerative diseases, including Alzheimer's disease, Amyotrophic lateral sclerosis and Parkinson's disease [2,4]. These characteristics include: 1) functional failure that precedes cell loss, 2) axon degeneration that precedes cell soma loss and 3) induction of neuroinflammatory responses by glial cells [2-4].

Cytokine signaling is a key component of the glial response in glaucoma and several cytokines are implicated as mediators of RGC survival, including Tumor Necrosis Factor alpha [5,6] and the Interleukin-6 family of cytokines [6-9]. Chemokines are a subset of cytokines that activate and recruit peripheral leukocyte populations to sites of infection or injury [10,11]. Like pro-inflammatory cytokines, several chemokines are implicated in glaucoma, particularly in the anterior segment as it relates to modulation of IOP [12-16]. However, the involvement of chemokines in retinal responses to glaucomatous stressors is not well understood.

Chemokines are organized based on the position of cysteine residues, and their biological effects are mediated through seven transmembrane-spanning G-protein-coupled receptors (GPCRs) [11,17]. Various C-C motif chemokines, also known as β-chemokines, are expressed in ocular tissues [18], with the β-chemokine Ccl2 (also known as MCP-1) being the most widely studied [19-22]. In retina, Ccl2 was recently identified as a mediator of photoreceptor degeneration and monocyte recruitment in a rat model of light-induced retinal degeneration [23,24] as well as a neuroprotectant for RGCs in a rat model of laser-induced glaucoma [25]. A second β-chemokine, CCL5 (also known as RANTES), may play a role in infections of the eye and autoimmune-related degeneration of the retina [26,27]. Elsewhere in the CNS, CCL5 and its GPCRs are constitutively expressed by astrocytes, microglia, neurons and endothelial cells [28-32]. CCL5 signaling is also associated with chronic neurodegenerative diseases [32-35], where it is hypothesized to initiate and amplify neuroprotective mechanisms for long-term protection of neurons [29,33,36-39].

Here, we sought to characterize expression of CCL5 and its high-affinity GPCRs in murine retina and discern the potential relevance of CCL5 signaling to glaucoma. Using a variety of molecular and histological techniques, we found that CCL5 and its high-affinity receptors are constitutively expressed in the murine retina and are differentially induced by both aging- and IOP-related stressors associated with glaucoma. CCR5 appeared to be the predominant receptor for constitutive CCL5 signaling, but CCR3 may be particularly important for CCL5 signaling in the inner retina. In both constitutive and stressor-induced contexts, localization patterns for signaling machinery indicates likely relevance for CCL5 signaling in synaptic layers of retina, particularly the inner plexiform layer.

## Materials and Methods

### Animals

Male DBA/2J and age-matched control C57BL/6mice were both obtained at 1 month of age from Charles River Laboratories (Wilmington, MA) and individually housed for the duration of the experiments. CCL5-deficient mice (CCL5^-/-^; 4 month) were obtained from Jackson Laboratories (Bar Harbor, ME) and bred in-house at the Vanderbilt University animal facilities.

### Ethics statement

This study was conducted in accordance with regulations set forth in the ARVO Statement for the Use of Animals in Ophthalmic and Vision Research. Animal protocols were approved by the Institutional Animal Care and Use Committees of the Vanderbilt University Medical Center.

### IOP measurements – DBA/2 model of glaucoma

**Figure 1:**
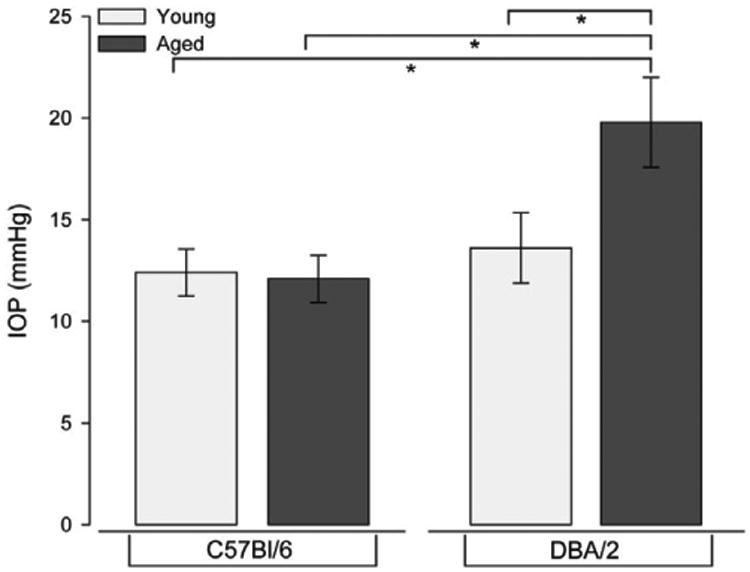
IOP measurements in age-matched C57Bl/6 and DBA/2 mice. Mean IOP measurement (mmHg) obtained in awake, behaving mice just prior to sacrifice. IOP increased by 35% with age in DBA/2 mice. Age did not alter IOP in C57Bl/6 mice. Error bars represent standard error and asterisks indicate p<0.05.

IOP measurements were obtained monthly in DBA/2 mice beginning at 2 months of age until sacrifice at 8 months of age. A minimum of ten readings per eye were obtained by tonometry in awake, behaving mice (Tonolab by Icare; Vantaa, Finland), as previously described [8,9,40]. Mean IOP measurements for all mice utilized in the study are depicted in Figure 1.

### Tissue procurement and preparation

For analysis of fresh retina tissue, mice were sacrificed by cervical dislocation followed by decapitation. Whole eyes were enucleated, flash-frozen and stored at -80°C until RNA isolation. For tissue procurement for paraffin-embedded and wholemount retina analyses, mice received an overdose of pentobarbital (200 mg/kg; Hospira, Inc., Lake Forest, IL) and were sacrificed by transcardial perfusion with PBS (Fisher Scientific; Pittsburg, PA) followed by 4% paraformaldehyde (PFA; Electron Microscopy Sciences, Hatfield, PA). Whole eyes were enucleated and post-fixed for one hour in PFA. For paraffin retina sections, eyes were paraffin-embedded and 6 μm serial sections of the entire globe were obtained. For wholemount preparations, whole retina was dissected from the eyecups and vitreous removed.

### Quantitative real-time polymerase chain reaction

Total RNA was isolated from retina with Trizol (Life Technologies; Grand Island, NY) and treated with DNase I, as previously described [8,9,41]. Quantitative PCR (qPCR) was performed by the Vanderbilt University Medical Center Genome Sciences Resource Microarray Core, as previously described. Briefly, only samples with quality control/quality assessment analysis (28S:18S ratios>0.9 and RNA integrity values>7) were used for qPCR experiments. Following reverse transcription with SuperScript II Reverse Transcriptase (Life Technologies), a cDNA clean-up was completed, using Agencourt Ampure XP PCR Purification kit, according to manufacturer's instructions (Beckman Coulter, Indianapolis, IN). To further confirm suitability of samples, a 1 μl of RNA from each sample was reserved to serve as a –RT negative control for gene-specific qPCR. Probe efficiencies and levels of mRNA for individual retinas were assessed using Taqman Gene Expression Master Mix (Applied Biosystems, Forest City, CA) and 1 μM of TaqMan probes specific for: CCL5 (Catalog# Mm01302427_m1); CCR1 (Catalog# Mm00438260_s1); CCR3 (Catalog# Mm00515543_s1); CCR5 (Catalog# Mm01963251_s1) and glyceraldehyde 3-phosphate dehydrogenase (gapdh; Catalog# Mm99999915_g1) on a 7900HT Fast Real-time PCR System in triplicate (Applied Biosystems). As indicated by probe efficiencies, the ΔCt method was used to determine expression (SDS software; Applied Biosystems). To calculate ΔCt, threshold of cycle (Ct) values for each gene were subtracted by the Ct values for the control gene GAPDH in each sample. Samples and controls were run in triplicate.

### Quantification of CCL5 secretion using luminex technology

To quantify CCL5 in retinal lysates (5 retina/sample), soluble extracts were isolated using the ProteoExtract Trans-Membrane Protein Extraction Kit (cat# 71772-3; Millipore) and analyzed using the Milliplex™ Map Mouse Cytokine/Chemokine Magnetic Bead Panel immunoassay kit (cat# MCYTOMAG-70K; Millipore). Quality controls and standards were run concurrently with experimental samples. Each control, standard and sample was run in duplicate. Total protein concentrations were determined using a BCA Protein assay (cat# 23227; Termo-Fisher).

### Immunoblotting

Immunoblotting against CCR5, CCR3 and CCR1 was performed in fresh-frozen retina, as previously described [8,41]. Briefly, retinas were homogenized in 50 mM Tris, pH 6.8, 2% SDS, 10% glycerol, 100 mM DTT, 2 mM EDTA, 50 mM NaF, 0.2 mM Na_3_VO_4_, 0.25 mM PMSF, and protease inhibitor cocktail (Roche). Protein concentration was determined with a Bio-Rad protein assay (Bio-Rad, Hercules, CA). Samples (60-80 μg of protein) were prepared in denaturing buffer containing: 100 mM Tris-HCl, pH 6.8, 4% SDS, 20% glycerol, 0.2% bromophenol blue, and 200 mM dithiothreitol and separated by SDS-PAGE. Membranes were blocked for one hour using Odyssey TBS Blocking Buffer (Cat# 927-50000; Licor) followed by primary antibody incubation overnight at 4°C (Table 1). Following washes, membranes were incubated for 1 h in the appropriate fluorescent-conjugated, secondary antibodies (400 ng/ml; Molecular Probes). Bands were visualized on an infrared imaging system (Li-Cor, Lincoln, NE). Densitometry measurements were obtained by three independent experimenters, using Odyssey software (Li-Cor). Immunoblotting was performed in triplicate.

### Fluorescent *in situ* hybridization

To assess CCL5 mRNA, we performed a fluorescent *in situ* hybridization of CCL5 (catalog #VB1-11329-01) using the QuantiGene ViewRNA ISH Tissue Kit, according to manufacturer's instructions (Affymetrix, Santa Clara, CA). Briefly, paraffin sections from C57BL/6and CCL5^-/-^ mice were deparaffinized at 60°C for 1 hour, washed in xylene, and postfixed in 4% PFA. Tissue was incubated with 1× pretreatment solution at 95°C, followed by a protease digestion at 40°C for 10 minutes. Tissue was washed three times in PBS and incubated with alkaline phosphatase-conjugated CCL5 probes at 40°C for 2 h. Following the 2 h incubation, tissue was washed and placed in storage buffer overnight. Fluorescently labeled substrate (Fast Red) was used to detect CCL5 probes followed by immunohistochemistry with primary antibody (see below) against the Müller cell marker glutamine synthetase (GSyn; see below) a DAPI counterstain, and visualized using Nikon Ti microscope (Nikon Instruments) and analyzed using the NIS Element software program (Nikon Instruments).

### Immunohistochemistry

Paraffin-embedded Retinal Sections. To evaluate the expression of CCL5 and its receptors, immunohistochemistry (IHC) against CCL5, CCR1, CCR3 or CCR5 was performed on paraffin-embedded, longitudinal sections of retina, as previously described [8]. Briefly, sections were deparaffinized, re-hydrated and treated with 0.1% sodium borohydride (Fisher Scientific) to quench auto-fluorescence. Sections were incubated for 2 hours at room temperature in a blocking solution containing: 5% normal horse serum and 0.1% Triton-X in PBS. Sections were then incubated overnight at 4°C in primary antibody solution containing: primary antibodies against CCL5, CCR5, CCR3 or CCR1 as well as cell type-specific markers for horizontal cells, amacrine cells, rod bipolar cells, RGCs and Müller glia (Table 1), 3% normal horse serum and 0.1% Triton-X. Primary antibody binding was visualized with 2 hour incubation in secondary antibody solution containing: the appropriate fluorophore-conjugated secondary antibody (1:200; donkey anti-mouse IgG, donkey anti-goat IgG or donkey anti-rabbit IgG; JacksonImmuno, West Grove, PA), 1% normal horse serum and 0.1% Triton-X. Some sections were then counterstained with the nuclear stain 4,6-diamidino-2-phenylindole (DAPI; 50 μg/ml; Invitrogen; Grand, Island, NY). Digital images of fluorescent labeling were obtained on either an inverted confocal microscopy (Olympus, Center Valley, PA) at the Vanderbilt University Cell Imaging Core. Images were analyzed using Olympus Fluoview (Olympus) or a Nikon Ti microscope (Nikon Instruments; Nikon Instruments, Melville, NY) with a Roper Scientific black and white camera (Photometrics, Tucson, AZ) and analyzed using the NIS Element software program (Nikon Instruments). Incubations containing either isotype-specific controls and primary or secondary antibody only served as negative controls.

### Image analysis

Co-localization of labeling between CCR3 or CCR5 and cell type-specific markers was conducted in Image J. Scatterplots of fluorescent intensity across each image were generated with auto threshold determination *via* the Costes method. Verification and statistical assessment of co-localization was conducted by Fay method randomization, in which pixel co-localization in each channel was compared to those in at least 25 randomized image shifts. P value of 1.00 represents loss of co-localization in the randomized images, which indicates true co-localization of pixels in the original image. P values of less than 0.95 indicate retention of co-localization in randomized images at greater than 95% confidence, indicating the potential for erroneous co-localization of pixels between the two samples. Tresholded Mander's split co-localization coefficients were used to compare co-localization patterns of CCR3 and CCR5 with cell type-specific markers. For Mander's split co-localization coefficients, a value of zero equals no co-localization and a value of one equals complete co-localization. Values reported indicate the proportion of CCR3 or CCR5 labeling that co-localizes with the given cell type-specific marker.

### Statistical analysis

All statistical tests were performed with SigmaPlot (Systat Software Inc., San Jose, CA). For CCL5 secretion, mean pg/ml/μg total protein values were compared using Student's t-test following normality test validation. For qPCR, mean delta cycles above threshold (Ct) values of *CCR*1, *CCR*3 and *CCR*5 were normalized to and compared among groups using either a one-way analysis of variance with pair-wise multiple comparisons by Bonferroni test or one-sample T-test. For immunoblotting, densitometry measurements were compared across groups. Mander's split co-localization coefficients for cell type-specific markers were compared for CCR3 and CCR5 by one-way analysis of variance with pair-wise multiple comparisons by Bonferroni test. For all analyses, p ≤ 0.05 was considered statistically significant.

## Results

### CCL5 is constitutively expressed in healthy retina

**Figure 2:**
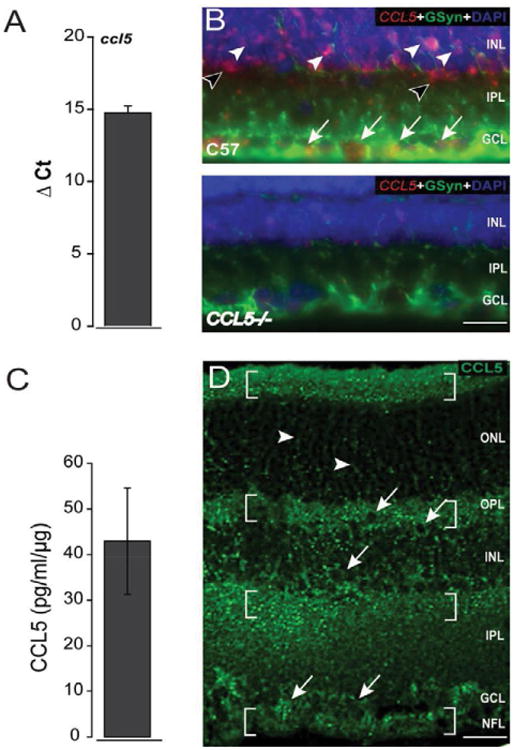
CCL5 is constitutively expressed in retina. A. Graphical representation of *CCL5* mRNA levels in naïve C57Bl/6retina (n=5), as measured by qPCR. The y-axis represents the average delta cycle of threshold (ΔCt) values for individual genes normalized to the control gene, *gapdh*. Error bars represent standard deviation. B. Representative micrograph of *CCL5* fluorescent *in situ* hybridization (red), co-immunolabeling with the Müller cell marker GSyn (green) and DAPI counterstain (blue) in longitudinal sections of naïve C57BL/6retina (n=12 retina sections from 3 mice). *CCL5* mRNA localizes mainly to the INL and GCL, where it is associated with GSyn^+^ Müller cell soma (filled arrowheads), RGC soma (filled arrows) and likely, amacrine cells (unfilled arrowheads). Retina sections from *CCL5*^-/-^ mice served as a control for specificity of the CCL5 probe (bottom panel). Scale=20 μm. C. Graphical representation of CCL5 protein expression (pg/ml/μg) in naïve C57Bl/6retina (n=15), as measured by Luminex Immunoassay. Error bars represent standard deviation. D. Representative confocal micrograph of CCL5 immunolabeling (green) in longitudinal sections of naïve C57BL/6retina (n=15 retina sections from 5 animals.) reveals strong punctate labeling in the synaptic layers (OPL and IPL) and near the outer limiting membrane (brackets). Additional labeling is consistent with Müller cell processes (arrowheads), RGC soma (arrows) as well as numerous somas throughout the INL. Scale=30 μm. ONL: Outer Nuclear Layer; OPL: Outer Plexiform Layer; INL: Inner Nuclear Layer; IPL: Inner Plexiform Layer; GCL: Ganglion Cell Layer; NFL: Nerve Fiber Layer.

We first examined expression and localization of CCL5 in retina from naïve C57Bl/6 mice. Using quantitative PCR, we found that CCL5 is constitutively expressed in healthy murine retina (Figure 2A). CCL5 mRNA localized to cell soma in the GCL (filled arrows) as well as both glutamine synthetase (GSyn)-positive soma (filled arrowheads) and GSyn-negative soma in the INL (Figure 2B). Specificity of our CCL5 probe was confirmed by negligible probe hybridization in retinal sections from ccl5-/- mice (Figure 2B, bottom panel). CCL5 protein was present at picogram quantities in naïve retina, as determined by ELISA (Figure 2C). Immunolabeling in longitudinal sections of retina demonstrated the presence of CCL5 protein in all layers (Figure 2D). Qualitatively, CCL5 labeling was concentrated in the synaptic layers of the retina as well as the NFL (Figure 2D). However, CCL5 also appeared to associate with cell soma of the upper and lower strata of INL and cell soma in the GCL as well as cell processes in the ONL (Figure 2D). Punctate labeling in both the OPL and IPL was concentrated near the outer and inner margins of INL, respectively, as well as just anterior to the ONL near the outer segments (Figure 2D).

**Figure 3:**
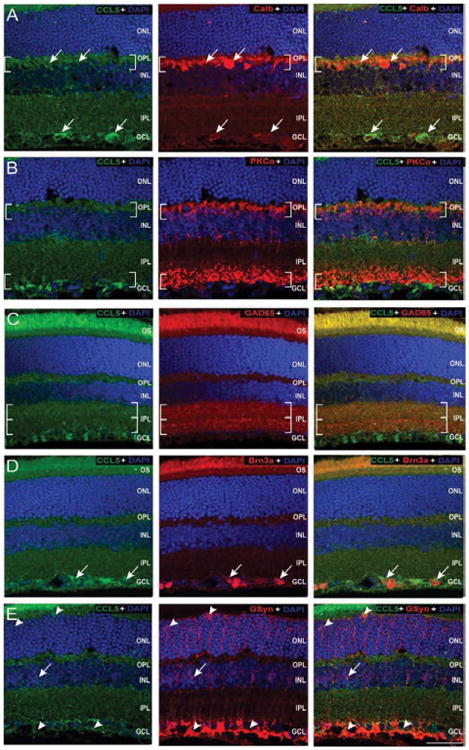
Constitutive CCL5 is closely associated with RGCs, Müller cells and inhibitory neurons. Representative confocal fluoromicrographs of retina sections from naïve C57BL/6retina immunolabeled with antibodies against CCL5 (green) and cell type-specific markers (red) for: horizontal cells and RGCs (calbindin; A), rod bipolar cells (PKCα; B), GABAergic amacrine cells (GAD65; C); RGCs (Brn3a; D), and Müller cells (GSyn; E). Immunolabeling was assessed in a minimum of 15 retina sections from 5 animals. Brackets indicate neuronal processes and terminals. Arrows indicate soma. Arrowheads indicate Müller cell processes and endfeet. Association between CCL5 and cell type-specific markers is identified by yellow labeling. Scale=40 μm. ONL: Outer Nuclear Layer; OPL: Outer Plexiform Layer; INL: Inner Nuclear Layer; IPL: Inner Plexiform Layer; GCL: Ganglion Cell Layer; NFL: Nerve Fiber Layer.

To identify cell types closely associated with the CCL5 protein, we co-immunolabeled longitudinal sections of C57Bl/6retina with antibodies against CCL5 and cell type-specific markers for horizontal cells, amacrine cells, rod bipolar cells, RGCs and Müller glia (Table 1). Confocal microscopy revealed close association of CCL5 labeling with: calbindin+ cell soma and processes in the OPL and INL (Figure 3A), calbindin+ soma in the GCL (Figure 3A), dendritic arbors and nerve terminals of PKC^α+^ cells in the OPL and IPL (Figure 3B), processes of GAD65+ cells in the IPL (Figure 3C), soma of Brn3a+ cells in the GCL (Figure 3D) and processes and endfeet of GSyn+ cells in the OPL, INL and NFL (Figure 3E).

#### High-affinity receptors for CCL5 are constitutively expressed in a layer-specific manner

**Figure 4:**
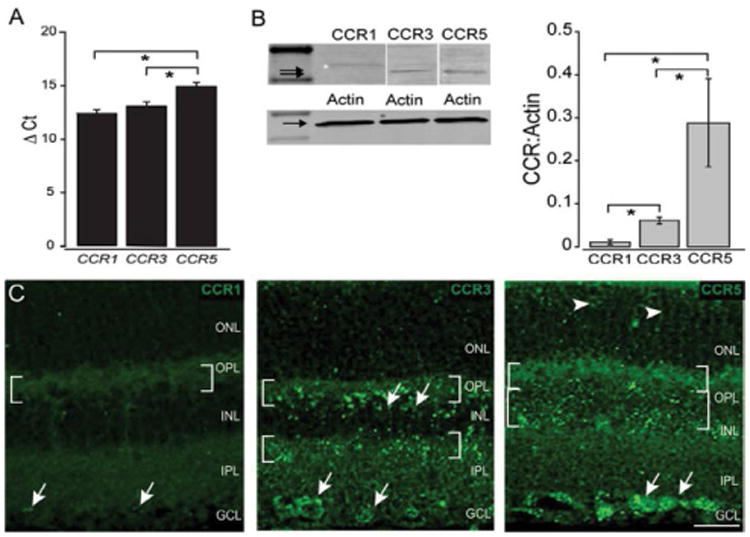
Constitutive expression of high-affinity CCL5 receptors in retina. A. Graphical representation *CCR5*, *CCR3* and *CCR1* mRNA levels in naïve C57Bl/6retina (n=15), as measured by qPCR. The y-axis represents the average delta cycle of threshold (ΔCt) values for individual genes normalized to the control gene, *gapdh*. Error bars represent standard deviation. Asterisks indicate p<0.05. B. Representative western blot of CCR5 (41 kDa), CCR3 (44 kDa) and CCR1 (41 kDa) expression in protein lysates from whole retina of naïve C57Bl/6mice (n=4; left). Arrows indicate expected molecular weight. Graphical representation of CCR5, CCR3 and CCR1 protein expression normalized to actin (right). Error bars represent standard deviation. Asterisks indicate p<0.05. C. Representative confocal micrographs of CCR1 (left), CCR3 (middle) and CCR5 (right) immunolabeling in longitudinal sections of naïve C57BL/6 retina. Immunolabeling was assessed in a minimum of 15 retina sections from 5 animals. Patterns of localization consistent with RGC soma in the GCL are evident for all three receptors (filled arrows). To varying degrees, CCR5 and CCR3 labeling also appears to localize to soma in the INL (arrowheads and brackets), Müller cell processes (filled arrowheads) and neuronal processes and terminals in the OPL and IPL (brackets). Scale=20 μm. ONL: Outer Nuclear Layer; OPL: Outer Plexiform Layer; INL: Inner Nuclear Layer; IPL: Inner Plexiform Layer; GCL: Ganglion Cell Layer; NFL: Nerve Fiber Layer.

To determine potential targets for CCL5 in murine retina, we examined whole retina gene expression as well as layer-specific protein expression of all three high-affinity receptors, CCR5, CCR3 and CCR1. Quantitative PCR in whole retina from naïve C57BL/6mice revealed that all three high-affinity receptors are constitutively expressed in murine retina, with greater expression of CCR5 than both CCR1 and CCR3 (p<0.01; Figure 4A). Immunoblotting for the three receptors exhibited the same trend, with CCR5 protein levels substantially higher than those of CCR3 and CCR1 (p<0.05; Figure 4B). Protein expression of CCR1 was particularly modest and significantly less than that of CCR3 (p<0.05; Figure 4B). Immunolabeling in longitudinal sections of retina confirmed constitutive expression of all three high-affinity receptors and revealed layer-specific differences in expression (Figure 4C). Consistent with protein levels obtained by immunoblotting, immunolabeling for CCR1 was markedly less intense than that of CCR5 and CCR3 (Figure 4C). Localization of CCR1 labeling was restricted to the INL and GCL (Figure 4C). In contrast, CCR3 labeling was present in the OPL, INL, IPL and GCL (Figure 4C). However, CCR3 labeling was most robust in the synaptic layers and the GCL, with labeling in the INL limited to discreet cell soma near the OPL (Figure 4C). Similarly, CCR5 immunolabeling was present in the OPL, INL and GCL (Figure 4C). Some CCR5 labeling was also noted in the ONL, but with far less intensity than the aforementioned layers (Figure 4C).

#### Association with the RGC-amacrine cell-bipolar cell microcircuit is common to all three Ccrs

**Figure 5:**
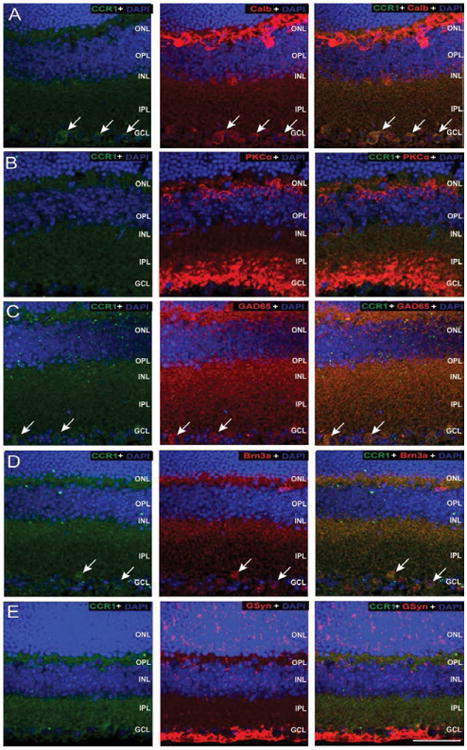
CCR1 is constitutively expressed by RGCs and amacrine cells in the GCL. Representative confocal fluoromicrographs of retina sections from naïve C57BL/6retina immunolabeled with antibodies against CCR1 (green) and cell type-specific markers (red) for: horizontal cells and RGCs (calbindin; A), rod bipolar cells (PKCα; B), GABAergic amacrine cells (GAD65; C); RGCs (Brn3a; D), and Müller cells (GSyn; E). Immunolabeling was assessed in a minimum of 15 retina sections from 5 animals. Co-localization of CCR1 with cell type-specific markers is identified by yellow labeling. CCR1 labeling is detected in the GCL where it associates the both Brn3a+ and GAD65+ soma (arrows). Scale=40 μm. ONL: Outer Nuclear Layer; OPL: Outer Plexiform Layer; INL: Inner Nuclear Layer; IPL: Inner Plexiform Layer; GCL: Ganglion Cell Layer; NFL: Nerve Fiber Layer.

**Figure 6:**
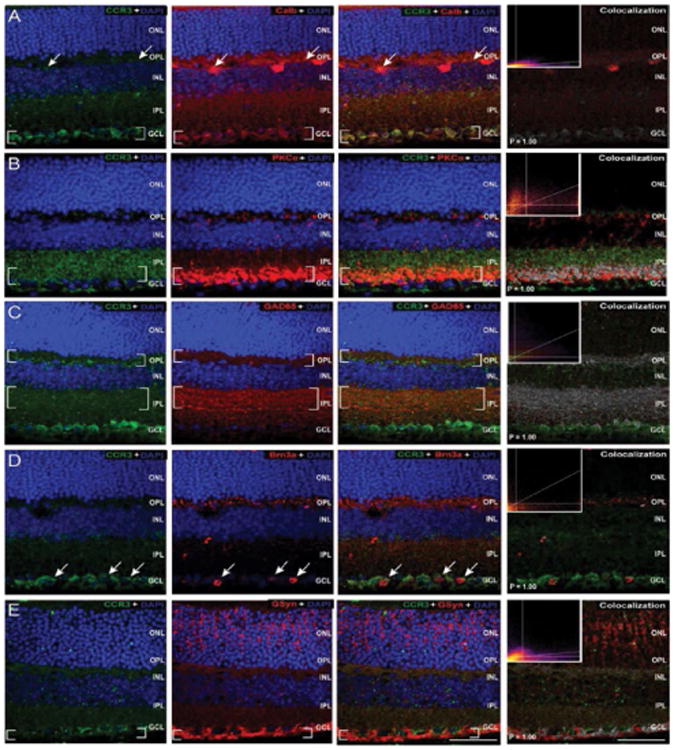
CCR3 is constitutively expressed by Müller cells as well as both excitatory and inhibitory neurons of the inner retina. Representative confocal fluoromicrographs of retina sections from naïve C57BL/6retina immunolabeled with antibodies against CCR3 (green) and cell type-specific markers (red) for: horizontal cells and RGCs (calbindin; A), rod bipolar cells (PKCα;B), GABAergic amacrine cells (GAD65; C); RGCs (Brn3a; D), and Müller cells (GSyn; E). Immunolabeling was assessed in a minimum of 15 retina sections from 5 animals. Co-localization of CCR5 with cell type-specific markers was determined by the Costes method with Fay randomization (P value) and depicted in white (right panels). Inserts depict colocalization frequency plots (right panels). CCR5 labeling is associated with calbindin+ RGCs in the GCL (A), PKCα^+^ bipoloar cell terminals in the IPL (B), GAD65+ soma and processes in the OPL and IPL (C), Brn3a+ soma in the GCL (D) and GSyn+ Müller cell endfeet in the GCL/NFL (E). Brackets indicate regions of co-localization. Scale=40μm. ONL: Outer Nuclear Layer; OPL: Outer Plexiform Layer; INL: Inner Nuclear Layer; IPL: Inner Plexiform Layer; GCL: Ganglion Cell Layer; NFL: Nerve Fiber Layer.

**Figure 7:**
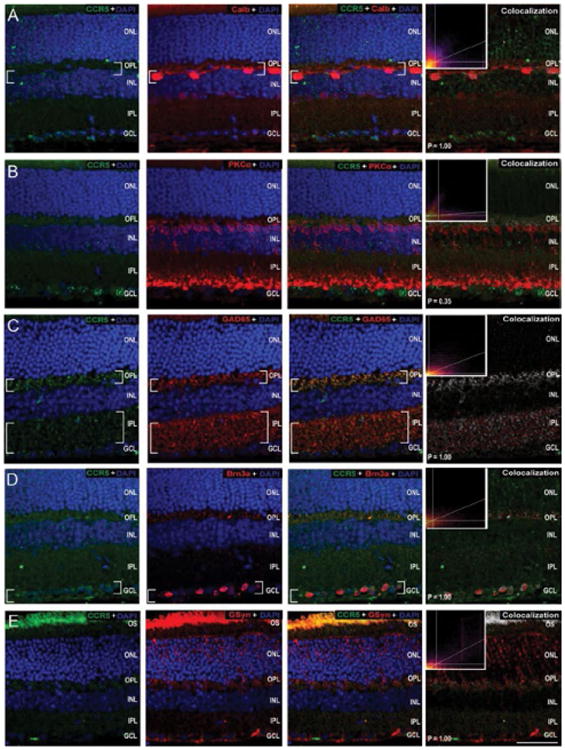
CCR5 is constitutively expressed by RGCs and inhibitory neurons in murine retina. Representative confocal fluoromicrographs of retina sections from naïve C57BL/6retina immunolabeled with antibodies against CCR5 (green) and cell type-specific markers (red) for: horizontal cells and RGCs (calbindin; A), rod bipolar cells (PKCα;B), GABAergic amacrine cells (GAD65; C); RGCs (Brn3a; D), and Müller cells (GSyn; E). Immunolabeling was assessed in a minimum of 15 retina sections from 5 animals. Co-localization of CCR5 with cell type-specific markers was determined by the Costes method with Fay randomization (P value) and depicted in white (right panels). Inserts depict colocalization frequency plots (right panels). CCR5 labeling is associated with calbindin+ horizontal cells in the OPL/INL (A), GAD65+ soma and processes in the OPL and IPL (C), Brn3a^+^ soma in the GCL (D) and GSyn+ Müller cell processes in the OPL (E). Brackets indicate regions of co-localization. Scale=40 μm. ONL: Outer Nuclear Layer; OPL: Outer Plexiform Layer; INL: Inner Nuclear Layer; IPL: Inner Plexiform Layer; GCL: Ganglion Cell Layer; NFL: Nerve Fiber Layer.

To identify specific cell types associated with CCR expression, we performed co-immunolabeling of all three CCRs with cell type-specific markers (Table 1) for retinal neurons and Müller glia in whole eye sections from naïve C57Bl/6mice. The sparse nature of CCR1 labeling versus robust labeling of cell type-specific markers precluded accurate assessment of co-localization *via* the Costes method. As such, our assessment is limited to qualitative association. We found that CCR1 immunolabeling most associated with calbindin+, GAD65+ and Brn3a+ cells in the GCL (Figures 5A, 5C and 5D). No staining overlap was noted between CCR1 and either PKCα or GSyn (Figures 5B and 5E). As determined by co-localization frequencies and illustrated by the intensity of co-localized pixels, CCR3 immunolabeling was most strongly associated with PKCα+ post- and pre-synaptic terminals in the OPL and IPL (Figure 6B) and GAD65+ processes in the IPL (Figure 6C). Weaker association was noted with calbindin+ cells in the INL and GCL (Figure 6A), Brn3a+ cells in the GCL (Figure 6D) and GSyn+ Müller cells (Figure 6E). All associations were confirmed as co-localization with P values of 1.00 *via* the Costes method with Fay randomization. In contrast, CCR5 immunolabeling was most strongly associated with GAD65+ processes in the OPL and IPL (Figure 7C) and Brn3a+ cells in the GCL (Figure 7D). Weaker association was noted with calbindin+ cells in the OPL (Figure 7A) and Müller cell soma near the INL (Figure 7E). These associations were confirmed as co-localization with P values of 1.00 *via* the Costes method with Fay randomization. Despite the appearance of Ccr5 co-localization in the pre-synaptic terminals of PKCα+ bipolar cells, this association achieved a P value of only 0.35 *via* the Costes method with Fay randomization (Figure 7B). This indicates that pixels perceived as potentially co-localized (far right panel, Figure 7B) are not truly co-localized, but are likely in close proximity.

**Figure 8:**
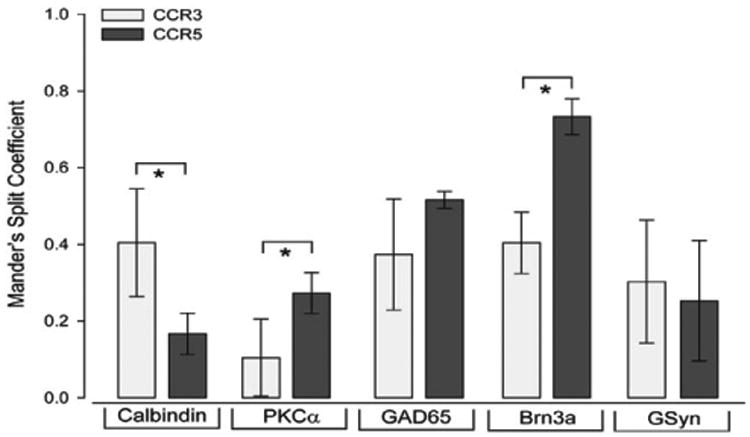
CCR3 and CCR5 are differentially expressed by inner retinal neurons. Graphical representation of thresholded Mander's split co-localization coeffcients in serial sections from the same retina co-immunolabeled with CCR3 or CCR5 and the following cell type-specific markers: horizontal cells and RGCs (calbindin), rod bipolar cells (PKCα), GABAergic amacrine cells (GAD65); RGCs (Brn3a), and Müller cells (GSyn). Coefficients represent the proportion of CCR3 or CCR5 pixels that co-localize with the given cell type-specific marker. Error bars represent standard deviation and asterisks indicate p<0.05.

To quantitatively compare expression profiles of CCR3 and CCR5, we measured the thresholded Mander's split co-localization coefficients of CCR3 and CCR5 with the same cell type-specific markers in serial sections from the same retina. For Mander's split co-localization coefficients, a value of zero equals no co-localization and a value of one equals complete co-localization. In Figure 8, coefficients represent the proportion of CCR3 or CCR5 pixels that co-localize with the given cell type-specific marker. Statistical comparison of Mander's split coefficients revealed significant differences in the co-localization patterns of CCR3 and CCR5 with calbindin, PKCα and Brn3a (Figure 8). Specifically, a greater proportion of CCR3 labeling co-localized with calbindin+ cells, as compared to CCR5 (p<0.05; Figure 8). In contrast, a greater proportion of CCR5 labeling co-localized with PKCα+ (p=0.01) and Brn3a+ (p<0.01) cells, as compared to CCR3 (Figure 8). Co-localization of CCR3 and CCR5 with GAD65+ and GSyn+ cells was proportionally similar (p>0.05; Figure 8).

#### Glaucoma-related stressors reduce expression of CCL5 signaling machinery in retina

**Figure 9:**
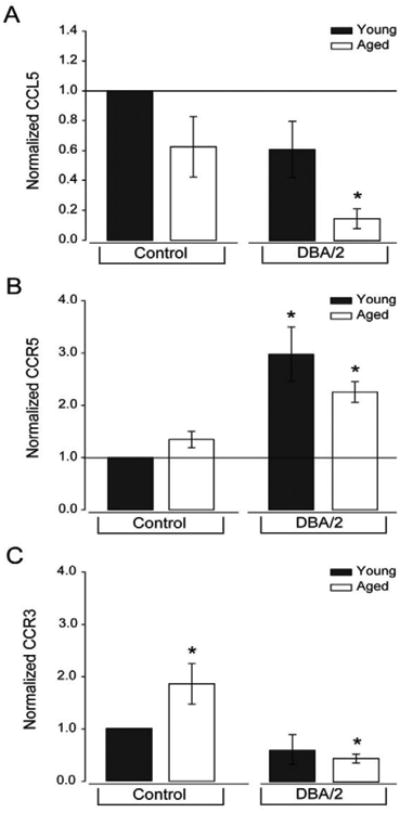
Glaucoma-related stressors in DBA/2 mice alter expression of CCL5 signaling machinery. A-C. Graphical representation of CCL5 (A), CCR5 (B) and CCR3 (C) protein expression in young (4 month) and aged (8 month) C57BL/6and DBA/2 retina (n=15 per group), as measured by Luminex immunoassay (CCL5) and immunoblotting (CCR5 and CCR3). Protein expression was calculated relative to total protein concentration for CCL5 and relative to actin expression for CCR5 and CCR3. Data is presented as expression normalized to young C57Bl/6mice. Error bars indicate standard deviation. Asterisks indicate p<0.05.

To determine whether glaucoma-related stressors alter expression of CCL5 signaling machinery, we examined protein expression and localization of CCL5, CCR5 and CCR3 in retina from age-matched C57Bl/6and DBA/2 mice. The DBA/2 mouse is an inbred strain that develops elevated IOP with age, resulting from mutations in the GPNMB and Tyrp1 genes [42,43]. Glaucomatous deficits in axonal transport and loss of RGCs as well as alterations in glial cell reactivity and cytokine signaling have been well-described in this model [8,9,40,44-47]. CCR1 was excluded from this analysis due to its sparse level of expression. Quantification of CCL5 expression in protein lysates from whole retina revealed that CCL5 expression was greatest in young C57BL/6mice and lowest in aged DBA/2 mice, which demonstrated a comparative 75% decrease in expression level (p<0.05; Figure 9A). CCL5 expression was more variable and similar in retina from both aged C57BL/6and young DBA/2 mice (Figure 9A). Changes in CCL5 expression between aged C57BL/6, young DBA/2 and young C57BL/6mice did not reach statistical significance (p>0.05 for both; Figure 9A). Both young and aged DBA/2 mice expressed higher levels of CCR5 than age-matched C57Bl/6retina (p<0.01 for both; Figure 9B). Normal aging in C57Bl/6mice and aging + elevated IOP in DBA/2 mice did not alter CCR5 expression (p>0.05; Figure 9B). In contrast, CCR3 expression increased by nearly 2-fold in aged C57BL/6mice, as compared to young C57BL/6mice (p < 0.05; Figure 9C). CCR3 expression appeared to decrease in young and aged DBA/2 mice, as compared to young C57BL/6mice (Figure 9C). However, this decrease only reached significance for aged DBA/2 mice, where CCR3 expression decreased by approximately 50% (p<0.05; Figure 9C).

**Figure 10:**
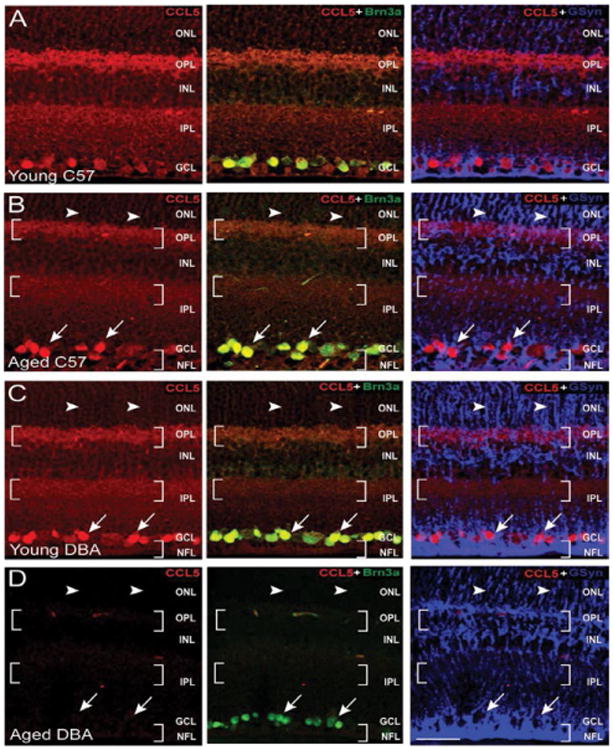
Glaucoma-related stressors in DBA/2 mice reduce Cl-5 expression associated with RGCs. Representative confocal fluoromicrographs of retina sections from young (4 month) and aged (8 month) C57BL/6(A,B) and DBA/2 (C,D) immunolabeled with antibodies against CCL5 (red) and cell type-specific markers for RGCs (Brn3a; green) and Müller cells (GSyn; blue). Immunolabeling was assessed in a minimum of 15 retina sections from 5 animals per group. Association between CCL5 and Brn3a or CCL5 and GSyn is evident by yellow and magenta labeling, respectively. Brackets indicate labeling in the OPL, INL and IPL. Arrowheads indicated labeling associated with Müller cell processes. Arrows indicate labeling associated with RGC soma. CCL5 labeling intensity and localization is most altered in aged DBA/2 retina, where labeling associated with RGCs is dramatically reduced. Scale=30 μm. ONL: Outer Nuclear Layer; OPL: Outer Plexiform Layer; INL: Inner Nuclear Layer; IPL: Inner Plexiform Layer; GCL: Ganglion Cell Layer; NFL: Nerve Fiber Layer.

Immunolabeling in whole eye sections were consistent with protein expression studies for CCL5, CCR5 and CCR3. For CCL5, the overall pattern of immunolabeling in young C57Bl/6, aged C57Bl/6and young DBA/2 retina was consistent with those depicted in Figure 2 (Figures 10A-10C). However, the intensity of CCL5 labeling, particularly in the synaptic layers, was lower in aged C57BL/6and young DBA/2 retina than that of young C57BL/6 retina (Figures 10A-10C). In aged DBA/2 retina, CCL5 labeling was markedly reduced and almost undetectable in all layers (Figure 10D). Although faint immunolabeling for CCL5 was detectable along the horizontal plane in the OPL and IPL, labeling was not detected in the GCL or along the vertical Müller cell projection (Figure 10D).

**Figure 11:**
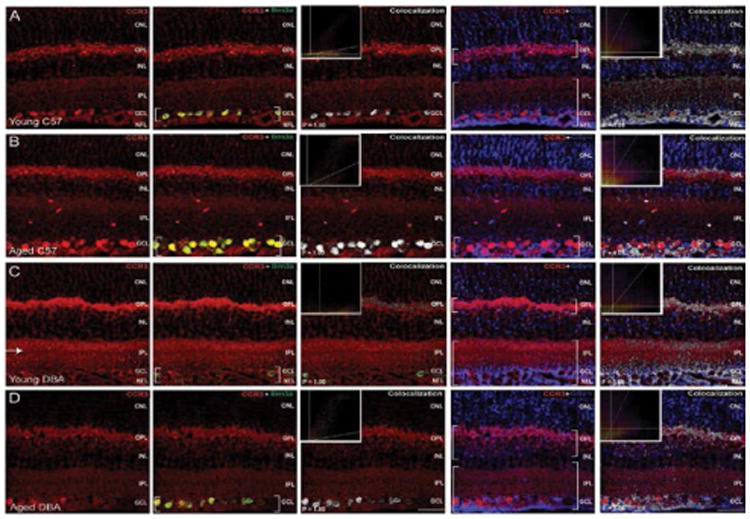
Glaucoma-related stressors modulate CCR3 expression, particularly in RGCs and Müller glia. Representative confocal fluoromicrographs of retina sections from young (4 month) and aged (8 month) C57BL/6(A,B) and DBA/2 (C,D) immunolabeled with antibodies against CCR3 (red) and cell type-specific markers for RGCs (Brn3a; green) and Müller cells (GSyn; blue). Immunolabeling was assessed in a minimum of 15 retina sections from 5 animals per group. Co-localization of CCR3 with cell type-specific markers was determined by the Costes method with Fay randomization (P value) and depicted in white (right panels). Inserts depict colocalization frequency plots (right panels). Aged C57Bl/6 exhibited increased CCR3 association with Brn3a+ RGCs, but decreased association with GSyn+ Müller glia. Conversely, young DBA/2 mice exhibited decreased CCR3 association with Brn3a+ RGCs, but increased association with GSyn^+^ Müller glia. Increased CCR3 labeling intensity in the IPL with demarcation of ON and OFF sublamina was also evident (arrow). In aged DBA/2 retina, CCR3 association with Brn3a+ RGCs and GSyn+ Müller glia was similar to young C57BL/6 and young DBA/2 retina, respectively. Brackets indicate regions of co-localization. Scale=30 μm. ONL: Outer Nuclear Layer; OPL: Outer Plexiform Layer; INL: Inner Nuclear Layer; IPL: Inner Plexiform Layer; GCL: Ganglion Cell Layer; NFL: Nerve Fiber Layer.

Like CCL5, patterns of CCR3 expression also shifted in aging and glaucomatous retina. As determined by co-localization frequencies and illustrated by the intensity of co-localized pixels, CCR3 labeling remained associated with Brn3a+ RGCs and GSyn+ Müller cells in all conditions (Figures 11A-11D). These associations were confirmed as co-localization with P values of 1.00 *via* the Costes method with Fay randomization. While co-localization was evident in all conditions, the magnitude of CCR3 labeling and the strength of its association with RGCs and Müller cells appeared to differ between conditions. Compared to young C57BL/6 retina, CCR3 co-localization increased with Brn3a, but decreased with GSyn, in aged C57BL/6 retina (Figures 11A and 11B). In young DBA/2 retina, CCR3 co-localization with Brn3a decreased substantially compared to all other conditions (Figure 11C). In contrast, CCR3 co-localization with GSyn appeared to increase in these mice relative to all other conditions (Figure 11C). The extent of co-localization between CCR3 and GSyn in young DBA/2 mice appeared most similar to that in aged DBA/2 mice (Figures 11C and 11D). Interestingly, young DBA/2 mice also exhibited increased intensity of CCR3 labeling in the IPL, where labeling demarcated ON and OFF sublamina established by bipolar cell terminals and RGC dendrites (arrow; Figure 11C). In aged DBA/2 retina, CCR3 co-localization with Brn3a was most similar to young C57BL/6retina (Figure 11D compare to 11A), while CCR3 co-localization with GSyn was most similar to young DBA/2 retina (Figure 11D compare to 11C).

**Figure 12:**
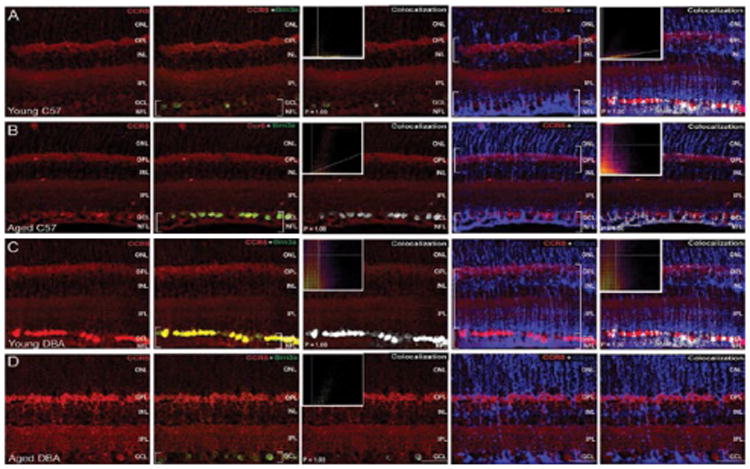
Glaucoma-related stressors increase CCR5 expression, particularly in the inner retina. Representative confocal fluoromicrographs of retina sections from young (4 month) and aged (8 month) C57BL/6(A,B) and DBA/2 (C,D) immunolabeled with antibodies against CCR5 (red) and cell type-specific markers for RGCs (Brn3a; green) and Müller cells (GSyn; blue). Immunolabeling was assessed in a minimum of 15 retina sections from 5 animals per group. Co-localization of CCR5 with cell type-specific markers was determined by the Costes method with Fay randomization (P value) and depicted in white (right panels). Inserts depict colocalization frequency plots (right panels). Intensity of CCR5 labeling in Brn3a^+^ RGCs appeared greater in aged C57Bl/6and young DBA/2 mice than in young C57BL/6or aged DBA/2 mice. In contrast, CCR5 labeling in GSyn^+^ Muller glia was absent in aged DBA/2 retina and markedly weaker in aged C57Bl/6 retina. Aged DBA/2 retina also exhibited increased CCR5 labeling in OPL, INL and IPL. Brackets indicate regions of co-localization. Scale=30 μm. ONL: Outer Nuclear Layer; OPL: Outer Plexiform Layer; INL: Inner Nuclear Layer; IPL: Inner Plexiform Layer; GCL: Ganglion Cell Layer; NFL: Nerve Fiber Layer.

Like CCR3, CCR5 remained associated with Brn3a+ RGCs across all conditions, as determined by co-localization frequencies and illustrated by the intensity of co-localized pixels (Figures 12A-12D). This association was confirmed as co-localization in all groups with P values of 1.00 *via* the Costes method with Fay randomization. However, the strength of CCR5 and Brn3a co-localization appeared markedly higher in aged C57Bl/6and young DBA/2 mice (Figures 12B and 12C) than in young C57BL/6or aged DBA/2 mice, which demonstrated similar degrees of co-localization (Figures 12A and 12D). Like Brn3a, GSyn association with CCR5 was confirmed as co-localization (P values of 1.00) for young C57Bl/6, aged C57Bl/6and young DBA/2 retina (Figures 12A-12C). However, the strength of this co-localization appeared markedly reduced in aged C57Bl/6retina (Figure 12B). In contrast, no co-localization was noted between GSyn and CCR5 in aged DBA/2 retina (Figure 12D). Interestingly, aged DBA/2 retina did exhibit increased intensity of CCR5 immunolabeling in the OPL, INL and IPL, with changes in the OPL and INL being most apparent (Figure 12D).

**Figure 13:**
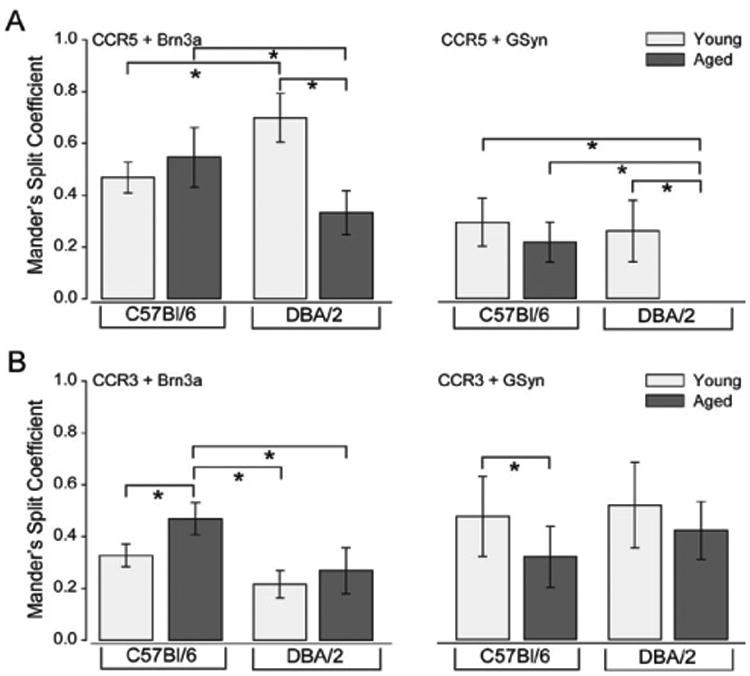
Glaucoma-related stressors differentially alter CCR3 and CCR5 expression in RGCs and Müller glia. Graphical representation of thresholded Mander's split co-localization coefficients in serial sections from the same retina co-immunolabeled with Brn3a, GSyn and CCR3 or CCR5. Coefficients represent the proportion of CCR3 or CCR5 pixels that co-localize with the given cell type-specific marker. Error bars represent standard deviation and asterisks indicate p<0.05.

To quantitatively compare changes in expression profiles of CCR3 and CCR5 associated with glaucoma-related stressors, we measured the thresholded Mander's split co-localization coefficients of CCR3 and CCR5 with Brn3a and GSyn in each condition. For Mander's split co-localization coefficients, a value of zero equals no co-localization and a value of one equals complete co-localization. In Figure 13, coefficients represent the proportion of CCR3 or CCR5 pixels that co-localize with Brn3a (left) or GSyn (right). Statistical comparison of Mander's split coefficients revealed that the localization pattern for CCR3 changed most dramatically in aged C57BL/6 retina (Figure 13A). Specifically, the proportion of CCR3 labeling co-localized with Brn3a was highest in aged C57Bl/6 retina (p< 0.05 for all). In contrast, the proportion of CCR3 labeling co-localized with GSyn was reduced in aged C57Bl/6 retina, as compared to young C57Bl/6 retina (p< 0.05; Figure 13A). Unlike CCR3, the localization pattern for CCR5 changed most dramatically in DBA/2 retina (Figure 13B). The proportion of CCR5 labeling associated with Brn3a was highest in young DBA/2 retina (p<0.05 for all), while the proportion associated with GSyn was lowest in aged DBA/2 retina (p<0.05 for all; Figure 13B).

## Discussion

Here we examined the potential relevance of the β-chemokine CCL5 in glaucoma. Through a series of expression and localization studies, we found that CCL5 and its high-affinity receptors are constitutively expressed in murine retina and are differentially induced by both aging- and IOP-related stressors associated with glaucomatous optic neuropathy.

Constitutively, CCL5 localizes to all layers of the retina, with the potential to influence a variety of neuronal and glial cell types. Based on localization patterns, CCL5 expression appears particularly relevant in the inner retina and synaptic layers. CCL5 associates with Müller cells and RGCs as well as synaptic connections between horizontal cells and bipolar cells in the OPL and amacrine cells, bipolar cells and RGCs in the IPL. Based on expression levels, CCR5 appears to be the predominant receptor for constitutive CCL5 signaling. However, localization studies reveal that CCR3 may be particularly important for mediating CCL5 signaling in the inner retina. CCR1 expression was detectable, but minute, with sparse localization in the inner retina. Although CCR3 and CCR5 are associated with similar cell types in the inner retina, co-localization studies with cell type-specific markers revealed preferential localization of CCR3 to calbindin+ cells and preferential localization of CCR5 to brn3a+ cells and PKCα+ cells. Calbindin is expressed by horizontal cells and some RGCs, while brn3a and PKCα are expressed by RGCs and rod bipolar cells, respectively. Although the functional significance of constitutive CCL5 signaling in retina is not yet clear, constitutive signaling in brain is associated with both neuronal development [48,49] and neurotransmission, particularly of glutamatergic systems [50,51]. The latter is of particular interest with respect to our findings, as our localization studies revealed strong association of CCL5 machinery with the RGC-amacrine cell-bipolar cell microcircuit. There is the potential that CCL5 may play a role in structural maintenance of and/or modulation of glutamatergic neurotransmission of this microcircuit.

To determine whether CCL5, like the β-chemokine Ccl2 [24,25], is involved in retinal neurodegeneration and specifically in glaucomatous degeneration of RGCs, we examined CCL5 expression and localization in the DBA/2 model of glaucoma. Analysis of protein expression indicated a dramatic decrease in CCL5 production in aged DBA/2 retina. CCL5 expression also appeared lower, but with significant variability in aged C57Bl/6and young DBA/2 retina versus young C57Bl/6retina. Based on histological examination of CCL5 protein localization, CCL5 expression appeared to decrease most in OPL, INL and IPL for aged C57Bl/6and young DBA/2 retina, which appeared similar in localization patterns and labeling intensity. In contrast, CCL5 labeling decreased substantially throughout the retina, including around RGCs. These data suggest that aging alone is not sufficient to impact CCL5 production near RGCs, but the introduction of elevated IOP does substantially reduce CCL5 production near RGCs.

In addition to changes in overall expression, it is likely that the functional consequences of aging- and glaucoma-related CCL5 signaling differ from that of constitutive signaling. This is at least partially supported by dramatic variations in expression and localization of both CCR5 and CCR3 in stressed retina. CCR5 expression was greater in DBA/2 mice than C57Bl/6mice, regardless of age. However, neither aging nor elevated IOP altered CCR5 expression levels. In contrast, CCR3 expression increased nearly 2-fold with normal aging in C57Bl/6 mice, but decreased by approximately 50% with aging + elevated IOP in DBA/2 mice. While protein expression studies in whole retina suggest that CCR5 is not impacted by glaucoma-related stressors, localization studies indicated that glaucoma-related stressors alter cell type-specific expression. In particular, CCR5 expression is greatest in Brn3a+ RGCs in young DBA/2 retina and lowest in GSyn+ Müller glia in aged DBA/2 retina. The localization pattern for CCR5 in aged DBA/2 retina suggests that this decrease in expression by Müller glia is ofset by increased expression in other retinal cell types in the OPL, INL and IPL. Based on our localization studies in naïve retina, this is most likely horizontal, bipolar or amacrine cells. In contrast, CCR3 localization was most impacted by normal aging, which induced increased expression in Brn3a+ RGCs and reduced expression in GSyn+ Müller glia. This suggests that CCL5 signaling preferentially impacts neuronal populations in aging retina.

In chronic neurodegenerative diseases of the brain, such as Multiple sclerosis, Alzheimer's disease and HIV-dementia, CCL5 signaling is associated not only with its classical definition as a chemoattractant for monocytes [31-36], but also with the initiation of neuroprotective mechanisms in neurons [33]. For example, cortical cultures pretreated with CCL5 are protected against inflammation-induced cell death [33]. In retinal neurodegeneration, there is evidence that β-chemokines can serve in both capacities as well. In a rodent model of light-induced photoreceptor degeneration, Ccl2 is upregulated by Müller cells and inhibition of this Ccl2 signaling inhibits monocyte recruitment and subsequently, reduces photoreceptor cell loss [24]. In contrast, intravitreal injection of recombinant Ccl2 promotes survival of RGCs in a rodent model of laser-induced glaucoma [25]. It is unclear whether the functional consequences of pressure-induced CCL5 signaling will follow a classical route of chemokine function or serve a more non-traditional role. However, based on the diversity and abundance of CCL5 machinery in retina, it is likely that the functional consequences of CCL5 signaling will depend on a variety of factors, including source, target, concentration effects and spatiotemporal characteristics.

It is important to note that, in both constitutive and induced capacities, several cell types appear to express CCL5 and at least one CCR, including RGCs, amacrine cells, bipolar cells and Muller glia. This indicates that there are a variety of potential autocrine and paracrine mechanisms for CCL5 signaling within and between these cell types in both healthy and pathological conditions. That stressor-related changed the expression and localization profile of CCL5 and CCR suggests that a shift in the nature and directionality of these autocrine and paracrine signaling pathways may be particularly relevant for pathological roles of CCL5 in RGC degeneration.

## Figures and Tables

**Table 1:** Primary antibodies for immunoblotting and immunohistochemistry.

Target	Antibody	Concentration	Vendor
Brn3a	Goat anti-human Brn3a	4 μg/ml	Santa Cruz Biotechnologies
Calbindin	Mouse anti-human Calbindin	1 μg/ml	Santa Cruz Biotechnologies
CCL5	Goat anti-mouse CCL5	16.7 μg/ml	R&D Systems
CCR1	Rabbit anti-human CKR1	4 μg/ml	Santa Cruz Biotechnologies
CCR3	Rabbit anti-human CKR3	4 μg/ml	Santa Cruz Biotechnologies
CCR5	Goat anti-human CKR5	4 μg/ml	Santa Cruz Biotechnologies
GAD65/67	Goat anti-human GAD65/67	4 μg/ml	Santa Cruz Biotechnologies
Glutamine Syntase	Goat anti-human Glutamine Synthetase	0.8 μg/ml	Santa Cruz Biotechnologies
	Rabbit anti-human Glutamine Synthetase	0.8 μg/ml	Santa Cruz Biotechnologies
PKCα	Rabbit anti-mouse, rat, human PKCa	0.25 μg/ml	AbCam
